# Prognostic significance of Spinster homolog gene family in acute myeloid leukemia

**DOI:** 10.7150/jca.44766

**Published:** 2020-05-18

**Authors:** Wenhui Huang, Tingting Qian, Zhiheng Cheng, Tiansheng Zeng, Chaozeng Si, Chaojun Liu, Cong Deng, Xu Ye, Yan Liu, Longzhen Cui, Lin Fu

**Affiliations:** 1 Department of Hematology, The Second Affiliated Hospital of Guangzhou Medical University, Guangzhou, 510260, China; 2 Translational Medicine Center, State Key Laboratory of Respiratory Disease, The Second Affiliated Hospital, Guangzhou Medical University, Guangzhou 510260, China; 3 Guangdong Provincial Education Department Key Laboratory of Nano-Immunoregulation Tumor Microenvironment, The Second Affiliated Hospital of Guangzhou Medical University, Guangzhou, 510260, China; 4 Department of Pathology and Medical Biology, University Medical Center Groningen, University of Groningen, Groningen, Netherlands; 5 Information Center, China-Japan Friendship Hospital, Beijing, 100029, China.; 6 Yinfeng Gene Technology Co., Ltd.; No.1109, Gangxing 3 Rd,New and High-tech Zone, Jinan City, Shandong Province, 250102, China; 7 Department of Clinical laboratory, The Second Affiliated Hospital of Guangzhou Medical University, Guangzhou, 510260, China; 8 Translational Medicine Center, Huaihe Hospital of Henan University, Kaifeng, 475000, China.; 9 Department of Hematology, Huaihe Hospital of Henan University, Kaifeng, 475000, China

**Keywords:** Acute myeloid leukemia, Prognosis, *SPNS1*, * SPNS2*, *SPNS3*

## Abstract

Acute myeloid leukemia (AML) is a clonal and heterogeneous disease characterized by proliferation of immature myeloid cells, with impaired differentiation and maturation. Spinster homolog (SPNS) is a widely distributed transmembrane transporter, which assists sphingolipids in playing their roles through the cell membrane. However, the expression and clinical implication of the *SPNS* family has not been investigated in AML. From the Cancer Genome Atlas database, a total of 155 AML patients with complete clinical characteristics and *SPNS1-3* expression data were contained in our study. In patients who received chemotherapy only, high expressions of *SPNS2* and *SPNS3* had adverse effects on event-free survival (EFS) and overall survival (OS) (all* P*<0.05). However, in the allogeneic hematopoietic stem cell transplantation (allo-HSCT) group, we only found a significant difference in OS between the high and low *SPNS3* expression groups (*P*=0.001), while other *SPNS* members showed no effect on survival. Multivariate analysis indicated that high *SPNS2* expression was an independent risk factor for both EFS and OS in chemotherapy patients. The results confirmed that high expression of *SPNS2* and *SPNS3* were poor prognostic factors, and the effect of *SPNS2* can be neutralized by allo-HSCT.

## Introduction

Acute myeloid leukemia (AML) is a malignancy with malignant breeding of bone marrow precursor cells, and the function and production of the normal cells are restrained 1. AML always accompanies with specific gene variations, which can be served as the basis of its onset and effective treatment 2, 3. Some gene abnormalities have been identified as independent prognostic factors. For example, high expressions of *FUT3/6/7*,* PDK2/3*,* PAK3/7* and *NCALD* were proved as poor prognosis factors in AML 4-7. While high *FUT4* and *PAK2* expressers have longer EFS and OS after chemotherapy 4, 6. On account of the previous studies, there should be more research to explore the effect of gene expression on prognosis.

Spinster homolog (SPNS) is a protein stretching across cell membrane, with the function of transmembrane transporter. According to amino acid sequence homology analysis, SPNS members belong to major facilitator superfamily (MFS) 8, 9. Previous study reported that SPNS1 and the vacuolar-type H+-ATPase (v-ATPase) could regulate proper autolysosomal biogenesis with optimal acidification, which is closely associated to developmental senescence and survival 10. In addition, Yanagisawa et. al identified that SPNS1 was a favorable factor in Niemann-Pick type C disease (NPC) (-/-) cells 11. SPNS2 is notarized to be the physiologically functional Sphingosine 1-phosphate (S1P) transporters, S1P is an effective and biologically active signaling molecules, which can promote the development of cancer by regulating cell proliferation, survival, migration, vascularization and lymphoangiogenesis 12, 13. The relation between SPNS2 and S1P were previously found in animals, such as zebrafishand and mouse. Then Hisano et. al demonstrated that human SPNS2 can also transport S1P and its analogue, indicating *SPNS2* may participate in the progress of cancer adjustment 14. There were studies indicated that *SPNS3* involved in sphingolipid pathways to mediate airway hyperresponsiveness and mast cell activation in asthma patients 15. People have probed some fundamental effects of *SPNS3,* nevertheless still don't understand most of the roles that *SPNS3* play in human disorders. But the prognosis of *SPNSs* in AML has never been investigated.

Here we conducted a prognosis study to investigate the impact of the *SPNS* genes in AML patients. Our study disclosed the guiding significance of *SPNS* expression in prognosis of AML, high expression of *SPNS2* and *SPNS3* were poor prognosis in chemotherapy patients, and *SPNS3* was a poor indicator for OS in allo-HSCT patients.

## Subjects and Methods

### Patients

A total of 155 AML patients with complete clinical data and* SPNS* expression from The Cancer Genome Atlas (TCGA) database were included in this study (https://cancergenome.nih.gov/). Eighty-four patients underwent chemotherapy only, and 71 also received allogeneic hematopoietic stem cell transplantation (allo-HSCT). Clinical characteristics of AML were expounded, the end points of this study were event-free survival (EFS) and overall survival (OS). OS referred to the time from diagnosis to death for any reason or the last follow-up time. EFS refers to the time from diagnosis to the first event, such as relapse, death, etc. Clinical and molecular characteristics were expounded, including peripheral blood (PB), white blood cell (WBC) counts, PB blasts, bone marrow (BM) blasts, French-American-British (FAB) subtypes, and the frequencies of known recurrent genetic mutations. The informed consent of patients was obtained, and the study protocol was approved by the Washington University Human Studies Committee.

### Statistical analysis

The clinical and molecular characteristics of the patients were summarized using descriptive statistical methods. Data sets were described by median and/or range. The Mann-Whitney *U*-test was used as appropriate to compare numerical comparison and *χ^2^* test for comparison of categorical and numerical data between two groups. Survival rates were estimated using the Kaplan-Meier method and the log-rank test. The univariate and multivariate Cox proportional hazard models of EFS and OS were established using a limited backward elimination process. The statistical significance level was 0.05 for a two tailed test. All statistical analyses were performed using SPSS software 25.0, and GraphPad Prism software 7.0.

### Bioinformatic Analysis

The median expression of *SPNS2* or *SPNS3* was demanded in 84 patients with chemotherapy-only group. The patients were divided into two group according to the median expression of *SPNS2* or *SPNS3*, then take the expression of *SPNS2* and *SPNS3* minus the median expression of *SPNS2* and *SPNS3* respectively. Gglot2 was used to map *SPNS2* or *SPNS3* gene expression profiles in these 84 AML patients. The gene expression above the median level is high expression. The Rice Hmisc package rcorr function was employed to investigate the Pearson correlation coefficient of the gene expression matrix, then genes related to *SPNS2* or *SPNS3* expression were extracted (p<0.01, absolute correlation coefficient >0.3). And genes associated with *SPNS2* or *SPNS3* expression were performed by the KEGG (Kyoto Encyclopedia of Genes and Genomes) pathway enrichment analysis. An unsupervised clustering heat map was generated for the first enriched significant pathway gene expression of *SPNS2* or *SPNS3* using the R-package ComplexHeatmap.

## Results

### Prognostic significance of *SPNS* family in AML

**Fig 1:**
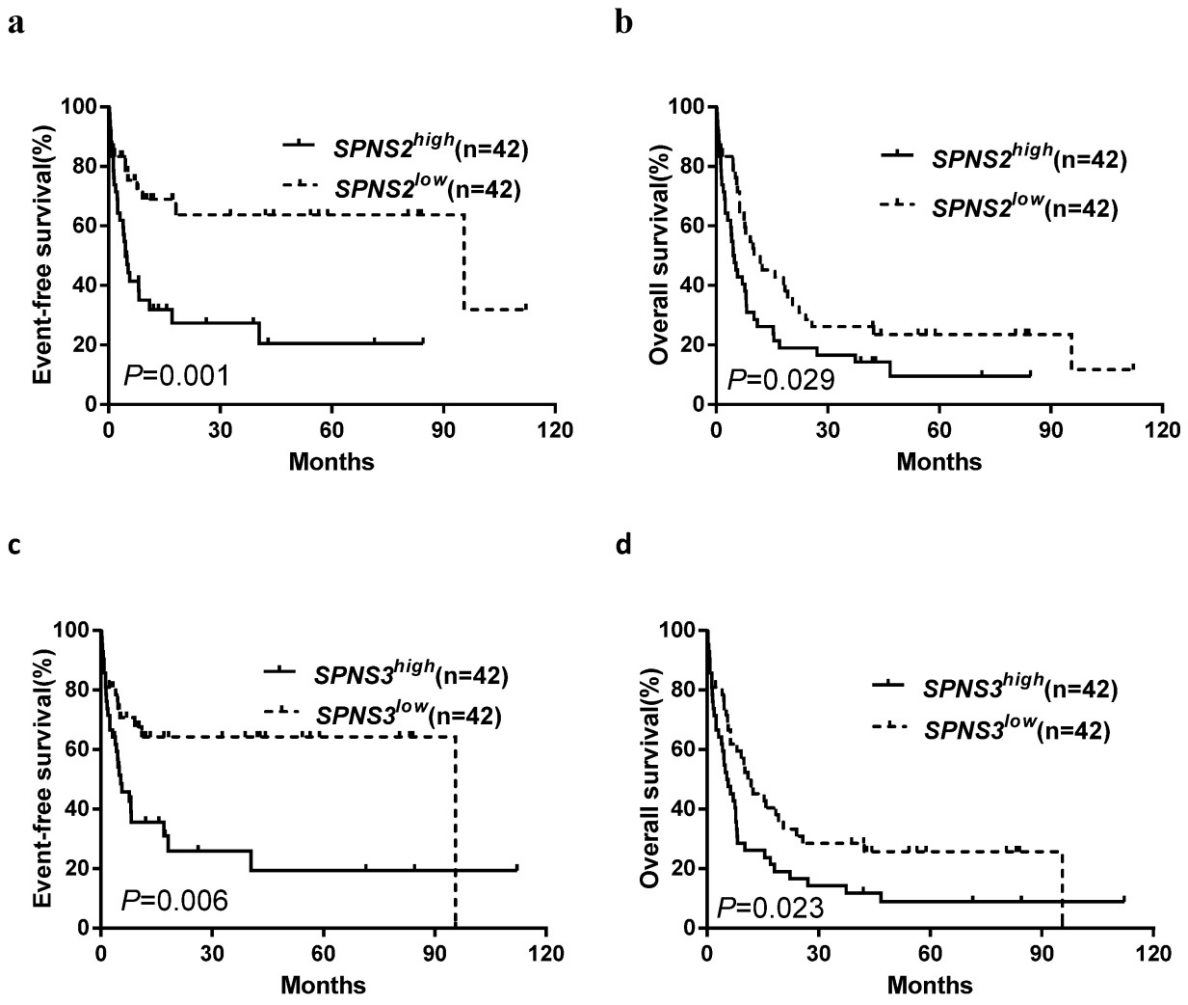
Kaplan-Meier curves of event-free survival (EFS) and overall survival (OS) in patients who received chemotherapy-only. **a, b** High *SPNS2* expressers had shorter EFS and OS than the low expressers. **c, d** High *SPNS3* expressers had shorter EFS and OS than the low expressers.

**Fig 2:**
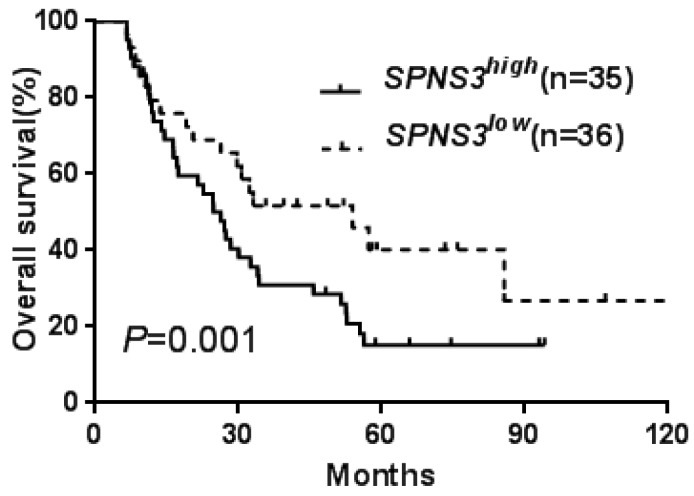
Kaplan-Meier curves of overall survival (OS) in patients who received transplantation treatment. High *SPNS3* expressers had shorter OS than the low expressers in allo-HSCT group.

All patients were divided into two groups according to median expression levels of the three *SPNS* members. The differences of EFS and OS between high and low expression subgroups were presented in Table 1. Kaplan-Meier analysis revealed that the chemotherapy-only patients with high *SPNS2* or *SPNS3* expression had an adverse effect on EFS and OS (all *P* < 0.05, Table 1, Fig. 1a-d). In the allo-HSCT group, high *SPNS3* expressers had a shorter OS than patients with low* SPNS3* expression (Table 1, Fig. 2).

### Clinical and molecular characteristics of the patients

As shown in Table 2, the clinical and molecular characteristics of high and low *SPNS2* and* SPNS3* expression subgroups in chemotherapy group were compared. In the* SPNS2*^high^ group, the group had more FAB-M1 (*P* < 0.001), fewer FAB-M4 (*P* = 0.004) and FAB-M5 patients (*P* = 0.003). No significant differences were observed in age and gender, WBC count, BM blasts, other FAB subtypes, risk stratification, frequencies of other genetic mutations *(FLT3-ITD*, *NPM1*, *DNMT3A*, *IDH1/IDH2*, *RUNX1*, *NRAS/KRAS*, *TET2*, and* TP53*) and relapse rates between the *SPNS2*^high^ and *SPNS2*^low^ groups. Compared with the *SPNS3*^low^ subgroup, *SPNS3*^high^ group had fewer patients with *RUNX1-RUNX1T1* karyotype (*P* = 0.026), and fewer patients with good-risk (*P* = 0.026). There are no remarkable differences were found in age and gender, WBC count, BM blasts, and PB blasts, FAB subtypes, other risk stratification, frequencies of other genetic mutations *(FLT3-ITD*, *NPM1*, *DNMT3A*, *IDH1/IDH2*, *RUNX1*, *NRAS/KRAS*, *TET2*, and* TP53*) and relapse rates between the two subgroups.

The clinical and molecular characteristics of high and low *SPNS3* expression in transplanted subgroup were shown in Table 3. Median age was 51 (range 18-72) years, with 19 cases older than 60 years. Thirty cases were man. The median WBC count, BM blasts, and PB blasts at diagnosis were 29.4 × 10^9^/L, 71%, and 48.5%, respectively. The primary FAB subtypes were M1, M2, and M4 (71.6%). Thirty-two patients had abnormal karyotypes. The proportion of good, intermediate, and poor-risk patients were 9.9, 59.2, and 29.6%, respectively. *NPM1* had the highest mutation frequency (n = 18, 25.4%), followed by *DNMT3A* (n = 17, 23.9%), *FLT3* (n = 17, 23.9%), *IDH1/2* (n = 17, 23.9%), *RUNX1* (n = 8, 11.3%), *NRAS/KRAS* (n =7, 9.9%), TET2 (n = 4, 5.6%), *TP53* (n = 4, 5.6%).Forty-eight patients had AML relapse. In regard to* SPNS3* expression, *SPNS3*^high^ group had more patients with normal karyotype (*P* = 0.017), more intermediate-risk (*P* = 0.016). No significant differences were observed in age and gender, WBC count, BM blasts and PB blasts, FAB subtypes, risk stratification, frequencies of other genetic mutations (*FLT3-ITD*, *NPM1*, *DNMT3A*, *IDH1/IDH2*, *RUNX1*, *NRAS/KRAS*, *TET2*, and* TP53*) and relapse rates between the *SPNS3*^high^ and *SPNS3*^low^ groups.

### Multivariate analysis of possible prognostic factors in the chemotherapy-only group and allo-HSCT group

In order to evaluating the prognostic effects of *SPNS2* and *SPNS3*, expression levels of *SPNS2/3* (high vs. low), age (≥60 vs. <60 years), PB blast count (≥20% vs. <20%), *FLT3-ITD* (positive vs. negative), and other common genetic mutations *(NPM1*,* DNMT3A*, *IDH1/IDH2*, *RUNX1* and *TET2*; mutated vs. wild) were selected for multivariate analysis (Table 4). In the chemotherapy-only group, we can conclude that high *SPNS2* expression was an independent poor factor for EFS and OS (*P* = 0.006, *P* = 0.048, respectively). And in the allo-HSCT group, high *SPNS3* expression (*P* = 0.002) and FLT3-ITD mutation (*P* = 0.035) were independent risk factors for OS.

### Bioinformatic analysis of *SPNS2* and *SPNS3* in chemotherapy-only group

**Fig 3:**
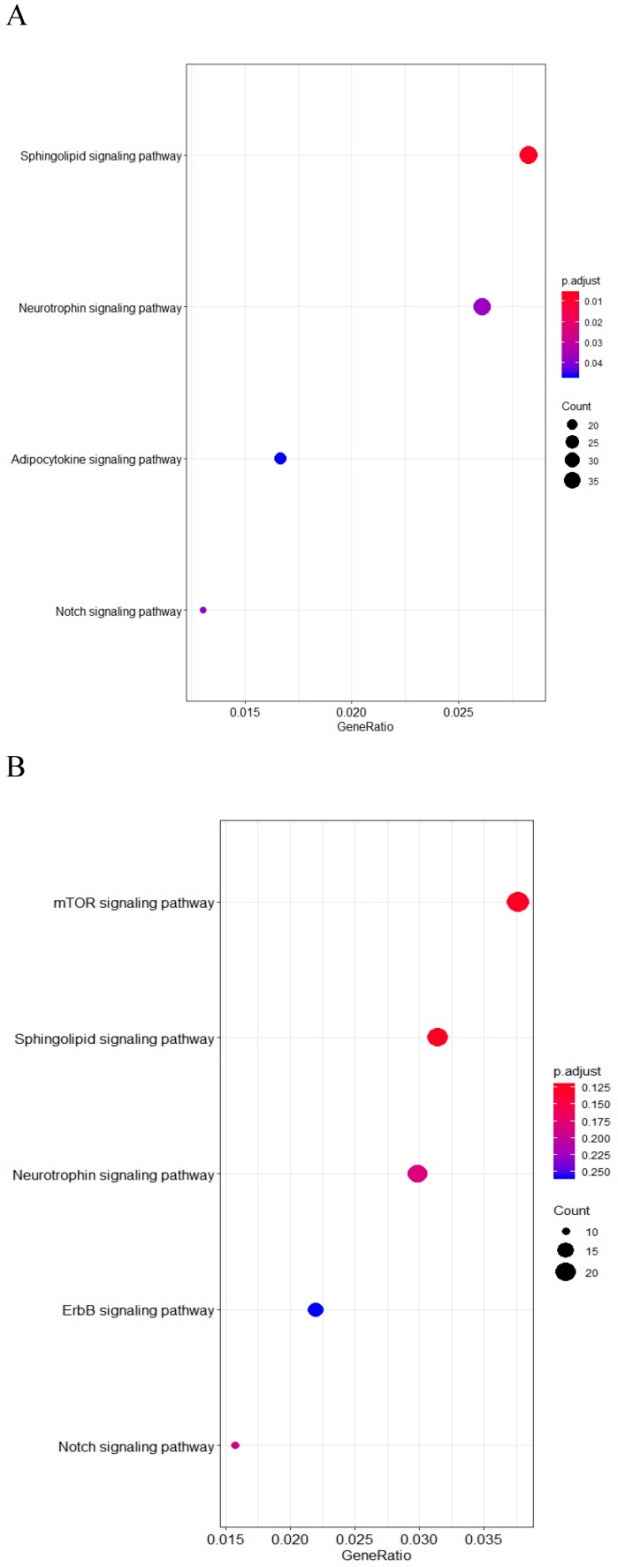
KEGG enrichment of Co-expression genes of *SPNS2* and *SPNS3*. A. Results of the KEGG pathway enrichment analysis associated with *SPNS2* expression. B. Results of the KEGG pathway enrichment analysis associated with *SPNS3* expression.

In order to explore the role of SPNS2 and SPNS3 in AML patients, we performed KEGG pathway enrichment analysis and mapped unsupervised clustering heat maps. The expressions of SPNS2 and SPNS3 were shown in Figure S1 and S2. There are 3100 positive and 1158 negative co-expression genes with *SPNS2* (Table S1). The results of the KEGG pathway enrichment analysis revealed that Neurotrophin, North, Adipocytokine and Sphingolipid signaling pathway were enriched in high *SPNS2* expressers (Fig 3A). And *SPNS2* was positive correlated with *AKT* and* TP53,* and negative associated with *PIK3R2,* all these genes belong to Sphingolipid signaling pathway (Fig S2, Table S2). According to the Table S3, 1941 positive and 440 negative co-expression genes with *SPNS3*. Different from *SPNS2*, patients who have high expression of *SPNS3* co-express with Sphingolipid, North, Neurotrophin, mTOR and ErbB signaling pathway (Fig 3B). Otherwise, the unsupervised clustering heat maps found that *SPNS3* was associated with *RPL* and *RPS* family, which expressed in Sphingolipid signaling pathway (Fig S4, Table S4).

## Discussion

In this study, we found high expression of *SPNS2* and *SPNS3* were poor prognostic factors in the patients who underwent chemotherapy only. Moreover, high expression of *SPNS3* was a negative prognosis factor for OS in allo-HSCT patients. *SPNS2* and *SPNS3* were independent dismal prognosis factors in chemotherapy and all-HSCT group respectively.

SPNS2*,* as a functional transporter of S1P, has been identified to be associated with many cancers. For example, previous study found that knockout *SPNS2* gene can worsen non-small cell lung cancer 16, another research illustrated *SPNS2* may play a role in inhibiting the development and progression of gastric cancer 17. However, another previous paper revealed that lacking of *SPNS2* can reduce the regulation ability of S1P on lymphocyte transport, leading to the reduced lymphocyte circulation in tissues, the proportion of T cells and NK cells then increased to kill the tumor cells more effectively 18. And SPNS2 can also promote the tumor growth via transporting S1P to extracellular environment 19.

In addition, the KEGG pathway enrichment manifested that *SPNS2* was closely related to the Sphingolipid signaling pathway, and a study revealed that Sphingomyelin pathway is a kind of Sphingolipid signaling pathway, which can lead to either cell proliferation and differentiation or to apoptosis. 20. The unsupervised clustering heat maps showed *SPNS2* can co-express with *AKT* in Sphingolipid signaling pathway*.* Previous passage talked a function material acid ceramide which participated in the Sphingolipid signaling pathway, can regulate cell apoptosis via AKT pathway. This suggested that there were some association between the AKT pathway and the Sphingolipid signaling pathway 21. However, the mechanism is unclear. In multiple analysis, *SPNS2* was proved to be an independent poor factor for the survival, indicating that *SPNS2* may related to carcinogenic function in AML, but the mechanism still needs to be further investigated.

*SPNS3* is an atypical Solute carriers (SLCs) of major facilitator superfamily (MFS) type 22, and it can mediate the progress of the apoptosis and autophagy in mammal 23-25. Autophagy-lysosome pathway (ALP) is one of the most important approach to eliminate abnormal proteins in human cells and there were some studies indicated that some genetic variation in autophagy-lysosome pathway plays a vital role in cancer development, such as lung cancer, gastric cancer, breast cancer, and renal cell carcinoma 26. From the KEGG genes enrichment we can see *SPNS3* mainly takes part in the Sphingolipid signaling pathway, and *SPNS3* may also develop its function via similar mechanism as *SPNS2* in AML 20. In our study, overexpressed *SPNS3* had a bad significance in both the chemotherapy group and the all-HSCT group. According to the above information, overexpression of *SPNS3* may regulate and control the progression, proliferation and differentiation of AML by autophagy. As patients with high *SPNS3* expression had bad survivals, *SPNS3* may can be used as predictor for AML patients in the future.

In multivariate analysis, *SPNS2* was proved to be an independent unfavorable prognosis factor for both EFS and OS in chemotherapy group, and *SPNS3* and *FLT3-ITD* were independent unfavorable prognosis factors for OS in the allo-HSCT group. Our result was along with the previous studies that *FLT3-ITD* was an adverse prognostic factor in AML 27, 28. And in our study, *SPNS2* and *SPNS3* were proved to be independent negative prognosis factors in chemotherapy and allo-HSCT respectively.

In summary, high expressions of *SPNS2* and* SPNS3* can indicate adverse prognosis in chemotherapy AML patients, and the prognosis effect of *SPNS2* can be overcome by allo-HSCT, and they might be used as predictors for AML patients in the future. Nevertheless, a larger sample size was needed to further validate our result and pathogenesis in AML still need further investigation.

## Supplementary Material

Supplementary figures and tables.

## Figures and Tables

**Table 1:** Comparison of EFS and OS between different expression levels of SPNS1-3

Variables	EFS			OS	
	*χ2*	*P*-value		*χ2*	*P*-value
Chemotherapy-only group					
* SPNS1* (high vs. low)	0.635	0.425		0.131	0.718
* SPNS2* (high vs. low)	11.465	0.001		4.784	0.029
* SPNS3* (high vs. low)	7.618	0.006		4.599	0.023
Allo-HSCT group					
* SPNS1* (high vs. low)	0.137	0.711		0.034	0.854
* SPNS2* (high vs. low)	0.033	0.856		1.760	O.185
* SPNS3* (high vs. low)	0.135	0.714		10.207	0.001

*Allo-HSCT* allogeneic hematopoietic stem cell transplantation, *EFS* event-free survival, *OS* overall survival

**Table 2:** Comparison of clinical and molecular characteristics in different SPNS2/3 expression groups among chemotherapy-only group.

Characteristics	*SPNS2*		*P*		*SPNS3*		*P*
	High (n=42)	Low (n=42)			High (n=42)	Low (n=42)	
Age (years), median (range)	67.5 (25-88)	66 (22-81)	0.975^a^		66 (33-88)	67 (22-81)	0.982^a^
Age group, *n* (%)			0.814^b^				0.814^b^
<60 years	12 (28.6)	14 (33.3)			14 (33.3)	12 (28.6)	
≥60 years	30 (71.4)	28 (66.7)			28 (66.7)	30 (71.4)	
Gender, *n* (%)			0.662^b^				0.382^b^
Male	21 (50.0)	24 (57.1)			25 (59.5)	20 (47.6)	
Female	21 (50.0)	18 (42.9)			17 (40.5)	22 (52.4)	
WBC (×10^9^/L), median (range)	14.8 (0.7-297.4)	14.6 (1.9-131.5)	0.862^a^		16.5 (0.7-134.4)	13.3 (1.0-297.4)	0.943^a^
BM blasts (%), median (range)	73.5 (32-99)	69.5 (30-95)	0.455^a^		74 (30-98)	68 (32-99)	0.785^a^
PB blasts (%), median (range)	49.5 (0-98)	7.5 (0-90)	0^a^		38 (0-97)	17.5 (0-98)	0.149^a^
FAB subtypes, *n* (%)							
M0	6 (14.3)	1 (2.4)	0.109^b^		3 (7.1)	4 (9.5)	1.000^b^
M1	18 (42.9)	2 (4.8)	0^b^		13 (31.0)	7 (16.7)	0.200^b^
M2	13 (31.0)	8 (19.0)	0.314^b^		9 (21.4)	12 (28.6)	0.615^b^
M4	4 (9.5)	16 (38.1)	0.004^b^		12 (28.6)	8 (19.0)	0.443^b^
M5	1 (2.4)	11 (26.2)	0.003^b^		4 (9.5)	8 (19.0)	0.350^b^
M6	0 (0.0)	1 (2.4)	1.000^b^		1 (2.4)	0 (0.0)	1.000^b^
M7	0 (0.0)	2 (4.8)	0.494^b^		0 (0.0)	2 (4.8)	0.494^b^
Cytogenetics, *n* (%)							
Normal	19 (45.2)	21 (50.0)	0.827^b^		22 (52.4)	18 (42.9)	0.512^b^
t(9;22)/BCR-ABL1	0 (0.0)	1 (2.44)	1.000^b^		0 (0.0)	1 (2.4)	1.000^b^
inv(16)/CBFβ-MYH11	1 (2.4)	5 (11.9)	0.202^b^		2 (4.8)	4 (9.5)	0.676^b^
Complex	7 (16.7)	5 (11.9)	0.520^b^		7 (16.7)	4 (9.5)	0.520^b^
11q23/MLL	1 (2.4)	2 (4.8)	1.000^b^		2 (4.8)	1 (2.4)	1.000^b^
t(8;12)/RUNX1-RUNX1T1	3 (7.1)	3 (7.1)	1.000^b^		0 (0.0)	6 (14.3)	0.026^b^
Others	11 (26.2)	6 (14.3)	0.277^b^		9 (21.4)	8 (19.0)	1.000^b^
Risk, n (%)							
Good	4 (9.5)	8 (19.0)	0.350^b^		2 (4.8)	10 (23.8)	0.026^b^
Intermediate	24 (57.1)	27 (64.3)	0.655^b^		27 (64.3)	24 (57.1)	0.655^b^
Poor	12 (28.6)	7 (16.7)	0.297^b^		11 (26.2)	8 (19.0)	0.603^b^
*FLT3, n (%)*			0.668^b^				0.447^b^
*FLT3-ITD*	6 (4.3)	9 (21.4)			8 (19.0)	7 (16.7)	
*FLT3-TKD*	9 (9.5)	3 (7.1)			5 (11.9)	2 (4.8)	
Wildtype	32 (76.2)	30 (71.4)			29 (69.0)	33 (78.6)	
*NPM1, n (%)*			0.641^b^				0.160^b^
Mutation	15 (35.7)	12 (28.6)			17 (40.5)	10 (23.8)	
Wildtype	27 (64.3)	30 (71.4)			25 (59.5)	32 (76.2)	
*DNMT3A, n (%)*			0.625^b^				0.141^b^
Mutation	13 (31.0)	10 (23.8)			15 (35.7)	8 (19.0)	
Wildtype	29 (69.0)	32 (76.2)			27 (64.3)	34 (81.0)	
*IDH1/IDH2, n (%)*			0.570^b^				1.000^b^
Mutation	9 (21.4)	6 (14.3)			7 (16.7)	8 (19.0)	
Wildtype	33 (78.6)	36 (85.7)			35 (83.3)	34 (81.0)	
*RUNX1, n (%)*			0.713^b^				1.000^b^
Mutation	5 (11.9)	3 (7.1)			4 (9.5)	4 (9.5)	
Wildtype	37 (88.1)	39 (92.9)			38 (90.5)	38 (90.5)	
*NRAS/KRAS, n (%*)			0.756^b^				0.756^b^
Mutation	5 (11.9)	7 (16.7)			5 (11.9)	7 (16.7)	
Wildtype	37 (88.1)	35 (83.3)			37 (88.1)	35 (83.3)	
*TET2, n (%)*			0.194^b^				1.000^b^
Mutation	8 (19.0)	3 (7.1)			5 (11.9)	6 (14.3)	
Wildtype	34 (81.0)	39 (92.9)			37 (88.1)	36 (85.7)	
*TP53, n (%)*			1.000^b^				0.520^b^
Mutation	5 (11.9)	6 (14.3)			7 (16.7)	4 (9.5)	
Wildtype	37 (88.1)	36 (85.7)			35 (83.3)	38 (90.5)	
Relapse/n (%)			0.261^b^				0.822^b^
Yes	13 (31.0)	19 (45.2)			15 (37.5)	17 (40.5)	
No	29 (69.0)	23 (54.8)			27 (64.3)	25 (59.5)	

*WBC* white blood cell, *BM* bone marrow, *PB* peripheral blood, *FAB* French American British ^a^Mann-Whitney *U*-test ^b^Chi-square test

**Table 3:** Comparison of clinical and molecular characteristics in different SPNS3 expression groups among allo-HSCT group

Characteristics	Total	*SPNS3*		*P*
		High (n=35)	Low (n=36)	
Age (years), median (range)	51 (18-72)	53 (21-65)	48.5 (18-72)	0.360^a^
Age group, *n* (%)				0.793^b^
<60 years	52 (73.2)	25 (71.4)	27 (75.0)	
≥60 years	19 (26.8)	10 (28.6)	9 (25.0)	
Gender, *n* (%)				0.153^b^
Male	30 (42.3)	18 (51.4)	12 (33.3)	
Female	41 (57.7)	17 (48.6)	24 (66.7)	
WBC (×10^9^/L), median (range)	29.4 (0.6-223.8)	19.6 (0.9-202.7)	30.7 (0.6-223.8)	0.441^a^
BM blasts (%), median (range)	71 (30-100)	69 (34-100)	75 (30-99)	0.982^a^
PB blasts (%), median (range)	48.5 (0-96)	57 (0-96)	43 (0-94)	0.118^a^
FAB subtypes, *n* (%)				
M0	9 (12.7)	5 (14.3)	4 (11.1)	0.735^b^
M1	23 (32.4)	14 (40.0)	9 (25.0)	0.211^b^
M2	18 (25.4)	11 (31.4)	7 (19.4)	0.285^b^
M3	1 (1.4)	0 (0.0)	1 (2.8)	1.000^b^
M4	13 (13.8)	4 (11.4)	9 (25.0)	0.220^b^
M5	4 (5.6)	1 (2.9)	3 (8.3)	0.614^b^
M6	1 (1.4)	0 (0.0)	1 (2.8)	1.000^b^
M7	1 (1.4)	0 (0.0)	1 (2.8)	1.000^b^
Cytogenetics, *n* (%)				
Normal	32 (45.1)	21 (60.0)	11 (30.6)	0.017^b^
t(9;22)/BCR-ABL1	2 (2.8)	0 (0.0)	2 (5.6)	0.493^b^
inv(16)/CBFβ-MYH11	5 (7.0)	1 (2.9)	4 (11.1)	0.357^b^
Complex	11 (15.5)	5 (14.3)	6 (16.7)	1.000^b^
11q23/MLL	3 (4.2)	1 (2.9)	2 (5.6)	1.000^b^
t(8;12)/RUNX1-RUNX1T1	1 (1.4)	0 (0.0)	1 (2.8)	1.000^b^
Others	16 (22.5)	6 (17.1)	10 (27.8)	0.396^b^
Risk, n (%)				
Good	7 (9.9)	1 (2.9)	6 (16.7)	0.107^b^
Intermediate	42 (59.2)	26 (74.3)	16 (44.4)	0.016^b^
Poor	21 (29.6)	8 (22.9)	13 (36.1)	0.300^b^
*FLT3, n (%)*				0.099^b^
* FLT3-ITD*	17 (23.9)	11 (31.4)	6 (16.7)	
* FLT3-TKD*	3 (4.2)	0 (0.0)	3 (8.3)	
Wildtype	51 (71.8)	24 (68.6)	27 (75.0)	
*NPM1, n (%)*				0.107^b^
Mutation	18 (25.4)	12 (34.3)	6 (16.7)	
Wildtype	53 (74.6)	13 (65.7)	30 (83.3)	
*DNMT3A, n (%)*				0.415^b^
Mutation	17 (23.9)	10 (28.6)	7 (19.4)	
Wildtype	54 (76.1)	25 (71.4)	29 (80.6)	
*IDH1/IDH2, n (%)*				0.173^b^
Mutation	17 (23.9)	11 (31.4)	6 (16.7)	
Wildtype	54 (76.1)	24 (68.6)	30 (83.3)	
*RUNX1, n (%)*				0.151^b^
Mutation	8 (11.3)	6 (17.1)	2 (5.6)	
Wildtype	63 (88.7)	29 (82.9)	34 (94.4)	
*NRAS/KRAS, n (%*)				0.484^b^
Mutation	7 (9.9)	4 (11.4)	3 (8.3)	
Wildtype	64 (90.1)	31 (88.6)	33 (91.7)	
*TET2, n (%)*				0.614^b^
Mutation	4 (5.6)	1 (2.9)	3 (8.3)	
Wildtype	67 (94.4)	34 (97.1)	33 (91.7)	
*TP53, n (%)*				1.000^b^
Mutation	4 (5.6)	2 (5.7)	2 (5.6)	
Wildtype	67 (94.4)	33 (94.3)	34 (94.4)	
Relapse/n (%)				0.614^b^
Yes	48 (67.6)	25 (71.4)	23 (63.9)	
No	23 (32.4)	10 (28.6)	13 (36.1)	

**Table 4:** Multivariate analysis of EFS and OS in chemotherapy-only group and allo-HSCT group.

Variables	EFS			OS	
	HR(95%CI)	*P*-value		HR(95%CI)	*P*-value
Chemotherapy-only group					
*SPNS2*	3.072 (1.378-6.851)	0.006		1.884 (1.006-3.526)	0.048
*SPNS3*	1.525 (0.737-3.156)	0.225		1.287 (0.728-2.276)	0.386
PB (≥20 vs. <20 × 10%)	0.680 (0.341-1.359)	0.275		0.653 (0.374-1.140)	0.134
*FLT3-ITD* (positive vs. negative)	0.889 (0.398-1.988)	0.775		0.863 (0.443-1.681)	0.666
*NPM1* (mutated vs. wild)	0.870 (0.398-1.948)	0.736		0.875 (0.473-1.617)	0.670
*DNMT3A* (mutated vs. wild)	1.265 (0.595-2.687)	0.541		1.626 (0.920-2.871)	0.094
*TET2* (mutated vs. wild)	0.476 (0.161-1.410)	0.180		0.619 (0.296-1.294)	0.202
Allo-HSCT					
*SPNS3*	1.092 (0.289-4.126)	0.897		2.789 (1.257-5.339)	0.002
Age (≥60 vs. <60 years)	0.740 (0.235-2.331)	0.607		1.061 (0.560-2.010)	0.856
PB (≥20 vs. <20 × 10%)	0.428 (0.149-1.227)	0.114		0.774 (0.404-1.481)	0.439
*FLT3-ITD* (positive vs. negative)	2.068 (0.450-9.508)	0.351		2.359 (1.062-5.240)	0.035
*NPM1* (mutated vs. wild)	0.597 (0.132-2.697)	0.502		0.502 (0.214-1.174)	0.112
*IDH1/IDH2* (mutated vs. wild)	1.456 (0.407-5.204)	0.563		0.789 (0.364-1.707)	0.547
*RUNX1*(mutated vs. wild)	2.275 (0.415-12.468)	0.344		1.529 (0.614-3.807)	0.361

*EFS* event-free survival, *OS* overall survival, *HR* hazard ratio, *CI* confidential interval, *PB* peripheral blood

## Abbreviations

AML: Acute myeloid leukemia
SPNS: Spinster homolog
EFS: Event-free survival
OS: Overall survival
MFS: Major facilitator superfamily
NPC: Niemann-Pick type C disease
S1P: Sphingosine 1-phosphate
TCGA: The Cancer Genome Atlas
allo-HSCT: Allogeneic hematopoietic stem cell transplantation
PB: Peripheral blood
WBC: White blood cell
BM: Bone marrow
FAB: French-American-British
KEGG: Kyoto Encyclopedia of Genes and Genomes
SLCs: Solute carriers
ALP: Autophagy-lysosome pathway
