# Supplementary material for: Prognostic significance of Spinster homolog gene family in acute myeloid leukemia
Source: J Cancer. 2020 May 18;11(15):4581–8. doi: 10.7150/jca.44766 (PMC7255376; doi:10.7150/jca.44766)
Supplement: Supplementary file 1 — Supplementary figures and tables. [file jcav11p4581s1.pdf]

SPNS2

Figure 1 Distribution of *SPNS2* expression level among 84 Chemotherapy-only patients.

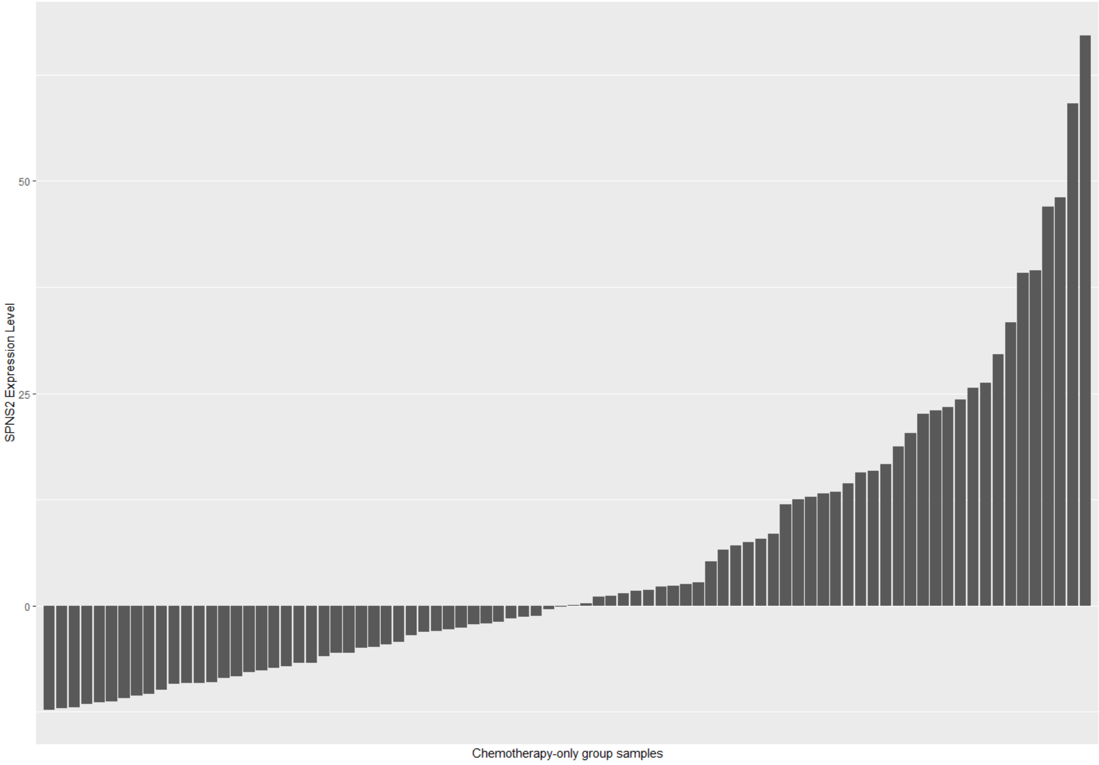

Table 1 Number of positive and negative co-expression genes with *SPNS2*

Positive: 3100 Negtive: 1158

Figure 2 Heatmap of genes that enriched in Sphingolipid signaling pathway

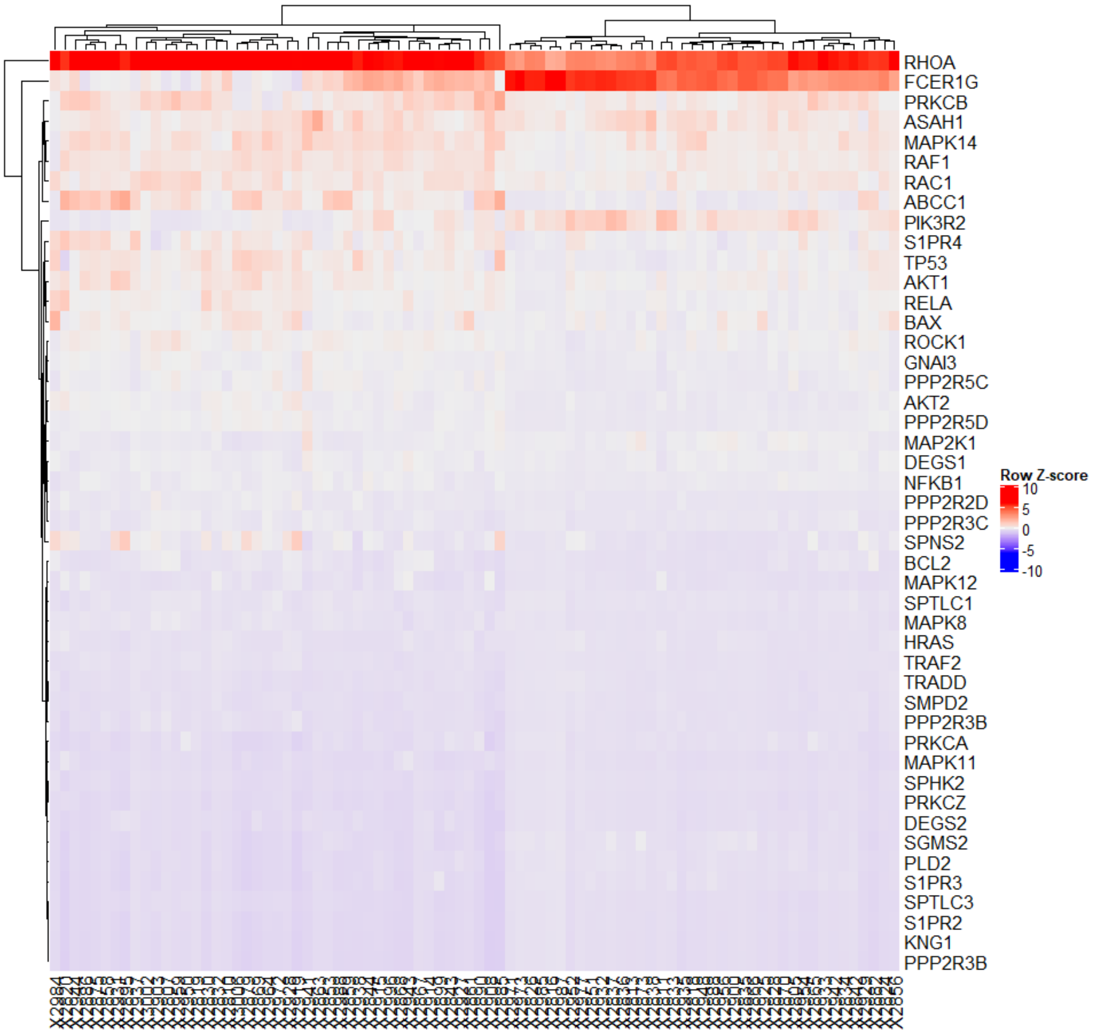

Table 2 Genes correlated with *SPNS2*

Gene Name	cor
ABCC1	0.523683
AKT1	0.30999
AKT2	0.624472
BAX	0.34092
BCL2	0.398772

DEGS2	0.41566
HRAS	0.412205
KNG1	0.401638
MAPK11	0.516029
MAPK12	0.344134
MAPK8	0.396831
PPP2R2D	0.384262
PPP2R3B	0.483439
PRKCZ	0.413783
RELA	0.514216
S1PR4	0.358807
SMPD2	0.405184
SPHK2	0.415152
SPTLC3	0.404723
TP53	0.43819
TRADD	0.33011
TRAF2	0.376445
ASAH1	-0.47634
DEGS1	-0.38313
FCER1G	-0.38244
GNAI3	-0.30349
MAP2K1	-0.4801

MAPK14	-0.44621
NFKB1	-0.36881
PLD2	-0.43392
PPP2R3C	-0.48975
PPP2R5C	-0.31493
PRKCA	-0.31925
RAC1	-0.32275
RAF1	-0.41965
RHOA	-0.38025
ROCK1	-0.38345
S1PR3	-0.40399
SGMS2	-0.35404
SPTLC1	-0.45194

SPNS3

Figure 3 Distribution of *SPNS3* expression level among 84 Chemotherapy-only patients.

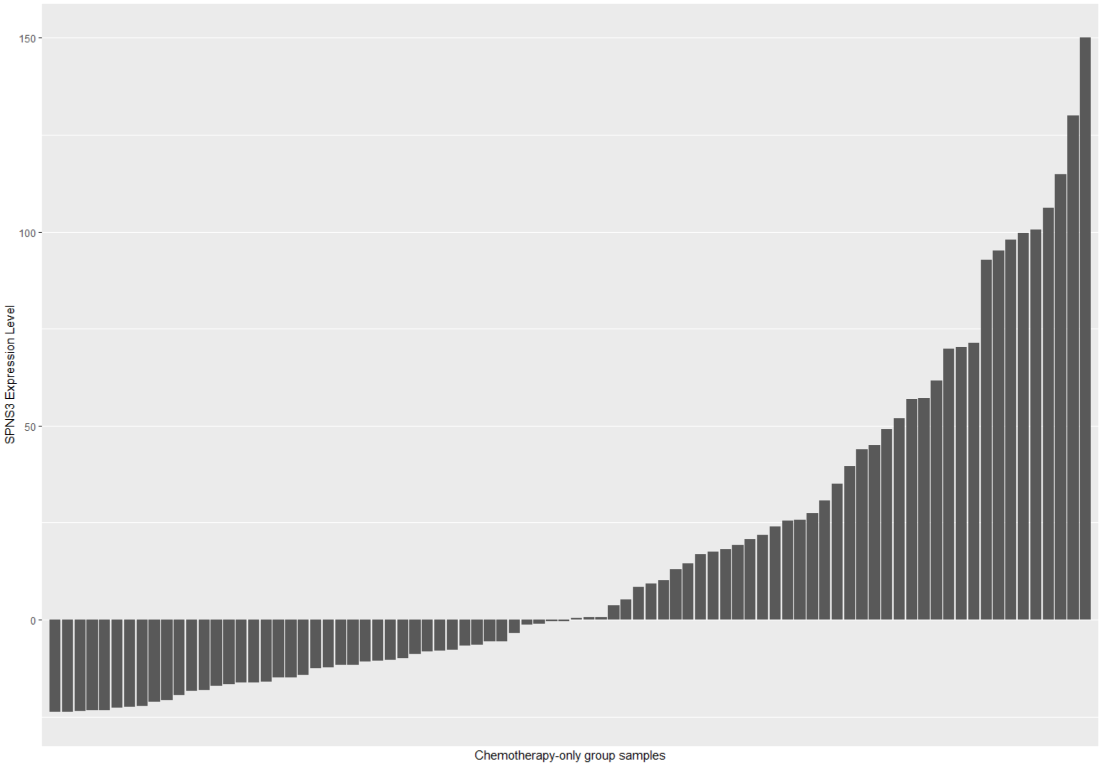

Table 3 Number of positive and negative co-expression genes with *SPNS3*

Positive:1491 Negtive440

Figure 4 Heatmap of genes that enriched in Sphingolipid signaling pathway

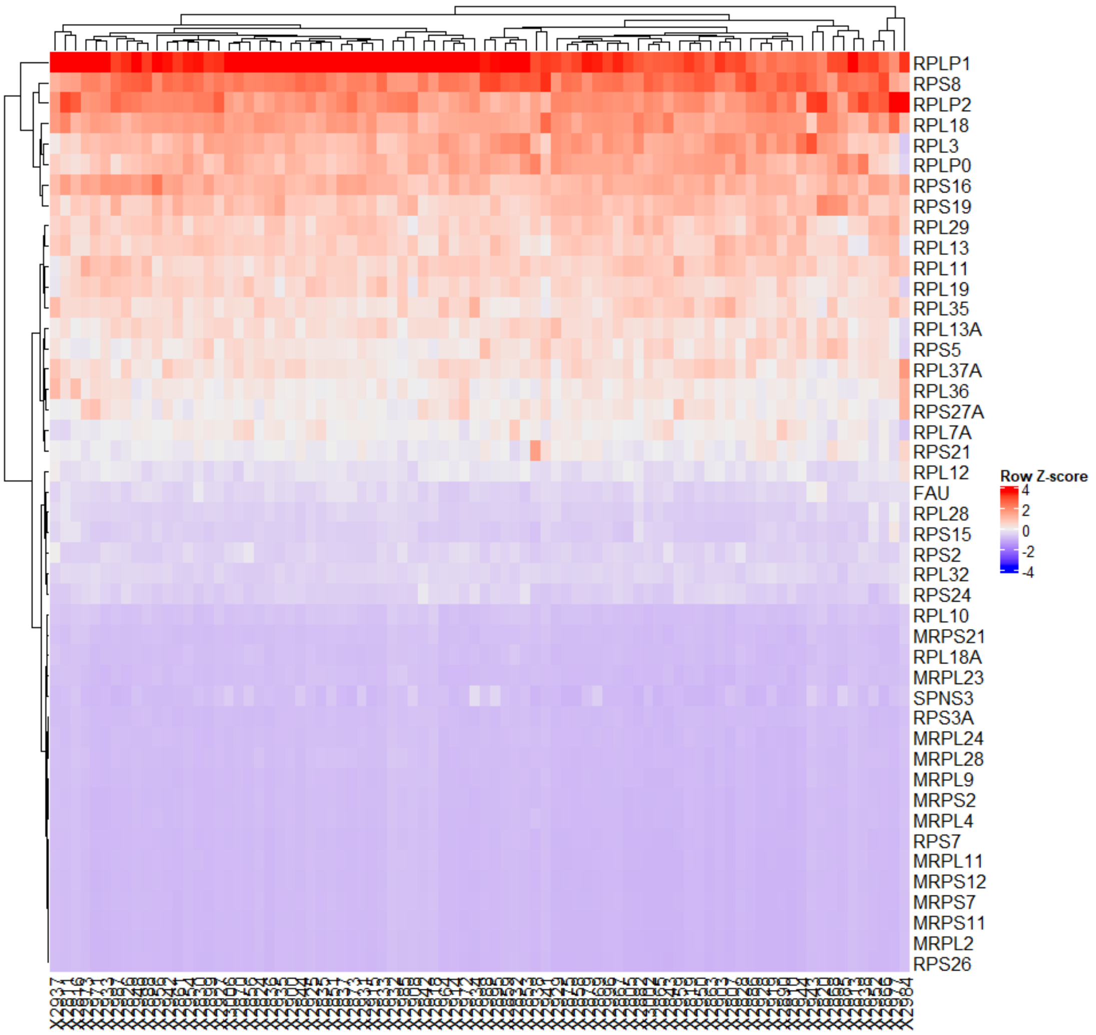

Table 4 Sphingolipid signaling pathway Genes correlated with SPNS2

genenames	cor
FAU	0.36401
MRPL11	0.447419
MRPL23	0.329104
MRPL24	0.301323
MRPL2	0.410111
MRPL4	0.348966
MRPL9	0.34174
MRPS12	0.358811
MRPS2	0.356368
MRPS7	0.36521

RPL10	0.365496
RPL11	0.340267
RPL12	0.328034
RPL13A	0.313535
RPL13	0.393324
RPL18A	0.334838
RPL18	0.35281
RPL28	0.332479
RPL29	0.354087
RPL32	0.339217
RPL35	0.303092
RPL7A	0.311082
RPLP0	0.314914
RPLP1	0.356117
RPLP2	0.356466
RPS15	0.334712
RPS19	0.394777
RPS2	0.351704
RPS5	0.33231
RPS7	0.312979
RPS8	0.341826
